# Development of a LAMP assay for detection of *Leishmania infantum* infection in dogs using conjunctival swab samples

**DOI:** 10.1186/s13071-015-0991-2

**Published:** 2015-07-15

**Authors:** Chun-hua Gao, Dan Ding, Jun-yun Wang, Dietmar Steverding, Xia Wang, Yue-tao Yang, Feng Shi

**Affiliations:** National Institute of Parasitic Diseases, Chinese Center for Disease Control and Prevention, Laboratory of Parasite and Vector Biology, Ministry of Public Health, National Center for International Research on Tropical Diseases, WHO Collaborating Centre for Malaria, Schistosomiasis and Filariasis, Shanghai, 200025 China; BioMedical Research Centre, Norwich Medical School, Norwich Research Park, University of East Anglia, Norwich, NR4 7TJ UK

**Keywords:** Loop-mediated isothermal amplification, Conjunctional swabs, Zoonotic visceral leishmaniasis, Asymptomatic canine reservoir host, *Leishmania infantum*

## Abstract

**Background:**

*Leishmania infantum* infections in dogs play a crucial role in the transmission of pathogens causing visceral leishmaniasis to humans in the Gansu province, northwest China. To be able to control zoonotic transmission of the parasite to humans, a non-invasive loop-mediated isothermal amplification (LAMP) assay to specifically detect *L. infantum* infections in dogs was developed.

**Methods:**

The primers used in the LAMP assay were designed to target kinetoplast DNA minicircle sequences of the *L. infantum* isolate MCAN/CN/90/SC and tested using DNA isolated from promastigotes of different *Leishmania* species. The LAMP assay was evaluated with conjunctional swab samples obtained from 111 and 33 dogs living in an endemic and a non-endemic region of zoonotic visceral leishmaniasis in the Gansu province, respectively. The LAMP assay was also compared with conventional PCR, ELISA and microscopy using conjunctional swab, serum and bone marrow samples from the dogs, respectively.

**Results:**

The LAMP assay detected 1 fg of *L. infantum* DNA purified from cultured promastigotes which was 10-fold more sensitive than a conventional PCR test using *Leishmania* genus-specific primers. No cross reaction was observed with DNA isolated from promastigotes of *L. donovani*, *L. major*, *L. tropica*, and *L. braziliensis*, and the *L. infantum* reference strain MHOM/TN/80/IPT1. The *L. infantum*-positive rates obtained for field-collected samples were 61.3 %, 58.6 %, 40.5 % and 10.8 % by LAMP, PCR, ELISA and microscopy, respectively. As only one out of the 33 samples from control dogs from the non-endemic region of zoonotic visceral leishmaniasis was positive by the LAMP assay and the PCR test, the observed true negative rate (specificity) was 97 % for both methods.

**Conclusion:**

This study has shown that the non-invasive, conjunctional swab-based LAMP assay developed was more sensitive in the detection of leishmaniasis in dogs than PCR, ELISA and microscopy. The findings indicate that the LAMP assay is a sensitive and specific method for the field surveillance of domestic dogs, particularly of asymptomatic canines, in ZVL-endemic areas in western China.

## Background

Leishmaniasis is a vector-borne parasitic disease of humans and other mammals caused by flagellates of the genus *Leishmania*. The protozoan parasites are transmitted by the bite of infected sandflies and live and multiply intracellularly in macrophages of their mammalian host. *Leishmania* parasites cause three different clinical forms of the disease in humans, classified as visceral leishmaniasis (VL), cutaneous leishmaniasis (CL) and mucocutaneous leishmaniasis (MCL). VL is considered to be the most lethal form of the disease causing, annually, an estimated 59,000 deaths and 2.4 million disability-adjusted life years (DALYs) [1]. The disease can be divided into two forms, namely zoonotic visceral leishmaniasis (ZVL) and anthroponotic visceral leishmaniasis (AVL). ZVL is widely distributed in the Mediterranean basin, Africa, Asia and Latin America and is caused by *L. infantum* [2–5].

VL is an important public health problem in China and is currently endemic in 61 counties in six provinces or autonomous regions in western China, including Xinjiang, Gansu, Sichuan, Shaanxi, Shanxi and Inner Mongolia [6]. Among them, Gansu, Sichuan, Shaanxi and Shanxi are ZVL endemic areas, with *Phlebotomus chinensis* as the vector for transmission of *L. infantum* between humans and dogs. Recent epidemiological studies have shown that the prevalence of canine leishmaniasis in western China is generally very high with over 50 % of dogs infected with *L. infantum* [7]. Elimination of domestic dogs in endemic areas has resulted in a dramatic reduction of human VL cases indicating that infected dogs play a crucial role as reservoir host [7, 8]. Diagnosis of canine leishmaniasis is difficult due to the wide spectrum of clinical manifestations and the lack of symptoms during the early stage of the infection [9–11]. Identification of infected dogs in endemic areas, in particular asymptomatic dogs, which constitute an important reservoir for the transmission of *L. infantum* to humans [12], is critical for the control of human VL. Thus, a reliable, accurate and rapid diagnostic test for the detection of canine leishmaniasis is needed in order to manage infected dogs and to avoid zoonotic transmission of the parasite to humans.

Serological and parasitological tests have limitations in the diagnosis of canine leishmaniasis, especially in early infected and asymptomatic dogs [13, 14]. On the other hand, molecular techniques have been shown to be very specific and sensitive in detection of *L. infantum* infections in dogs [7, 14–18]. However, as classical molecular methods like conventional polymerase chain reaction (PCR) require technically skilled staff and delicate equipment, they are not suitable for field studies in endemic regions. More recently, loop-mediated isothermal amplification (LAMP) was developed using DNA polymerase with strand-displacement activity along with two inner and two outer primers recognizing six separate target regions to rapidly amplify DNA with high specificity under isothermal condition [19]. This method combines the high sensitivity of a molecular diagnostic test with the possibility of carrying out the assay under field conditions with limited technical requirements.

Most molecular tests for diagnosis of leishmaniasis use blood, bone marrow, lymph node or skin samples [20]. However, these samples are obtained by invasive procedures. A non-invasive alternative is the collecting of conjunctival swab samples as a source of DNA [21]. In this study, a non-invasive, conjunctival swab LAMP assay for diagnosing *L. infantum* infection in dogs was developed and compared with a conventional PCR test using the same samples.

## Methods

### Ethics, consent and permissions

This study was reviewed and approved by the Ethical Review Committee of the National Institute of Parasitic Diseases, Chinese Center for Disease Control and Prevention in Shanghai. Oral informed consent was obtained from dog owners.

### Culturing of parasites

Five *Leishmania* species were used in this study (Table 1). Promastigotes of each species were grown in Novy-MacNeal-Nicolle medium (NNN medium) at 22-24 °C. After about 7 days of culture, promastigotes were harvested in stationary growth phase for DNA extraction.

**Table 1:** *Leishmania* species used in this study

Species	WHO code	Origin	Host	Form
*L. infantum*	MCAN/CN/90/SC	China (Sichuan)	dog	VL
*L. donovani*	MHOM/IN/80/DD8	India (Bihar)	human	VL
*L. infantum*	MHOM/TN/80/IPT1	Tunisia	human	CL
*L. major*	MHOM/SU/75/5ASKH	Turkmenistan	human	CL
*L. tropica*	MHOM/SU/73/K27	Azerbaijan	human	CL
*L. braziliensis*	MHOM/BR/75/M2903	Brazil (State of Pará)	human	CL

### Clinical samples

Sampling was carried out in November 2012 in Longnan city, Wudu district, Gansu province; an endemic area for ZVL. One-hundred-and-eleven dogs older than six months were included in this study. Before sampling, animals were subjected to clinical examination. Exfoliated epithelial cells were collected from the conjunctiva of the right and left eye of each dog using sterile cotton swabs manufactured for bacteriological isolation. The cotton tips were cut off and only the cotton parts were transferred to sterile tubes containing 200 μl of sterile saline, kept on ice and then stored at −20 °C until analyzed. Blood samples were collected from dogs in tubes without anticoagulant to obtain serum (1 ml). Serum samples were stored at −20 °C until analyzed. Bone marrow aspirates of each dog were obtained using a sterile biopsy needle and smeared on three slides. After drying, the smears were fixed with methanol and later stained with Giemsa for microscopic examination.

For negative controls, conjunctival swabs and serum samples were collected from 33 dogs living in the Zhang Xian County of Gansu province, a non-endemic area of ZVL. As this county is a high altitude area (>2500 m above sea level) with a yearly average temperature below 10 °C, sandflies do not normally occur and, therefore, dogs are usually not infected with *L. infantum*, For this reason, no bone marrow samples were taken from these dogs in order to avoid unnecessary distress and pain for the animals.

### DNA extraction

DNA of cultured promastigotes was isolated using phenol/chloroform extraction method as described in Schönian *et al*. [22]. DNA from conjunctival swabs was extracted by boiling the cotton parts in 200 μl of 0.9 % NaCl for 15 min followed by centrifugation at 10,000 g for 15 min at 4 °C.

### Loop-mediated isothermal amplification

**Fig. 1:**
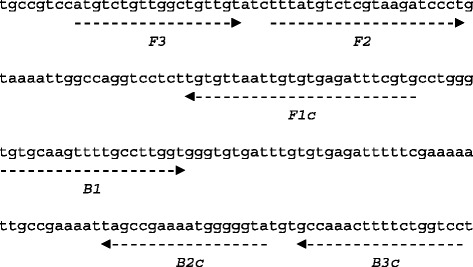
LAMP primer targeting *L. infantum* kinetoplast minicircle DNA. The partial sequence of *L. infantum* MCAN/CN/90/SC kinetoplast minicircle DNA and the location of the four primer, FIP (F1c-F2), BIP (B1-B2c), F3 and B3c are shown. Primer FIP consists of F1 complementary sequence and F2 direct sequence. Primer BIP consists of B1 direct sequence and B2 complementary sequence. The arrows indicate the direction of extension

LAMP primers were designed using PrimerExplorer software (http://primerexplorer.jp/e/) based on kinetoplast DNA (kDNA) minicircle sequence of the *L. infantum* isolate MCAN/CN/90/SC (Genbank, accession no. KC492147) (Table 2). The locations of the targeted sequences are shown in Fig. 1. The *L. infantum* specific LAMP assay was optimized for temperature and time using the strain MCAN/CN/90/SC. Different *Leishmania* species and strains (listed in Table 1) were used to determine the specificity of the LAMP assay.

**Table 2:** Nucleotide sequences of LAMP primers designed for detection of *L. infantum* MCAN/CN/90/SC kinetoplast minicircle DNA

Name	Length (bp)	Sequence
FIP (F1c-F2)	46	5’-CACGAAATCTCACACAATTAACACA- TTTATGTCTCGTAAGATCCCT-3’
BIP (B1-B2c)	38	5’-TGTGCAAGTTTTGCCTTGGT- TACCCCCATTTTCGGCTA-3’
F3	18	5’-ATGTCTGTTGGCTGTTGT-3’
B3c	18	5’-GGACCAGAAAAGTTTGGC-3’

The LAMP assay was carried out in 25 μl of reaction mixture containing 40 pmol of each FIP and BIP primers, 5 pmol of each F3 and B3 primers, 1.4 mM of each deoxynucleotide triphosphate, 0.8 M betaine, 20 mM Tris–HCl (pH 8.8), 10 mM KCl, 10 mM (NH_4_)_2_SO_4_, 8 mM MgSO_4_, 0.1 % Triton X-100, 8 units of *Bst* DNA polymerase large fragment (New England Biolabs, Ipswich, MA, USA), 1 μl calcein, and 1 μl DNA sample. The reaction mixture was placed in a Loopamp Real-time Turbidimeter LA-320C (Eiken Chemical Co, Ltd., Shanghai, China) and incubated at 64 °C for 60 min. The reaction was terminated by increasing the temperature to 80 °C. For confirmation, 3 μl of LAMP product was separated by electrophoresis on a 2 % agarose gel and visualized under UV light after staining with ethidium bromide (5 μg/ml).

The sensitivity of the LAMP assay was determined with 10-fold serially diluted DNA (10 pg to 0.1 fg) isolated from promastigotes of *L. infantum* MCAN/CN/90/SC. To compare the sensitivity of the LAMP assay with that of conventional PCR, the same dilutions were also tested by PCR.

When analyzing field samples, DNA extracted from conjunctival swabs from six dogs with symptomatic VL and confirmed *L. infantum* infection from Sichuan province served as positive controls. DNA samples extracted from conjunctival swabs from non-infected dogs were used as negative controls.

### Conventional polymerase chain reaction

For PCR, *Leishmania* genus-specific primers (RV1 (sense): 5’-CTTTTCTGGTCCCGCGGGTAGG-3’; RV2 (antisense): 5’-CCACCTGGCCTATTTTACACCA-3’ [23]) were used to amplify a 145-bp fragment of kDNA minicircle. The primers were synthesized by Shanghai Sangon Biological Engineering Technology & Service Co. Ltd. (Shanghai, China). PCR amplification was carried out as described previously [24]. PCR products were analyzed by electrophoresis on 2 % agarose gels.

### Microscopy

Methanol-fixed bone marrow smears were stained with Giemsa and examined under 100× oil-immersion lens for presence of *Leishmania* amastigotes. Two investigators examined independently at least 2000 microscopic fields of each smear.

### ELISA

An enzyme-linked immunosorbent assay (ELISA) was carried out as previously described using promastigote antigen isolated from cultured *L. infantum* MCAN/CN/90/SC [7].

### Statistical analysis

The *χ*^2^-test (http://www.quantpsy.org/chisq/chisq.htm) was used for comparing LAMP, PCR, ELISA and microscopy results. A *p* value less than 0.05 was considered to be statistically significant.

## Results

### Sensitivity of *L. infantum* LAMP assay

**Fig. 2:**
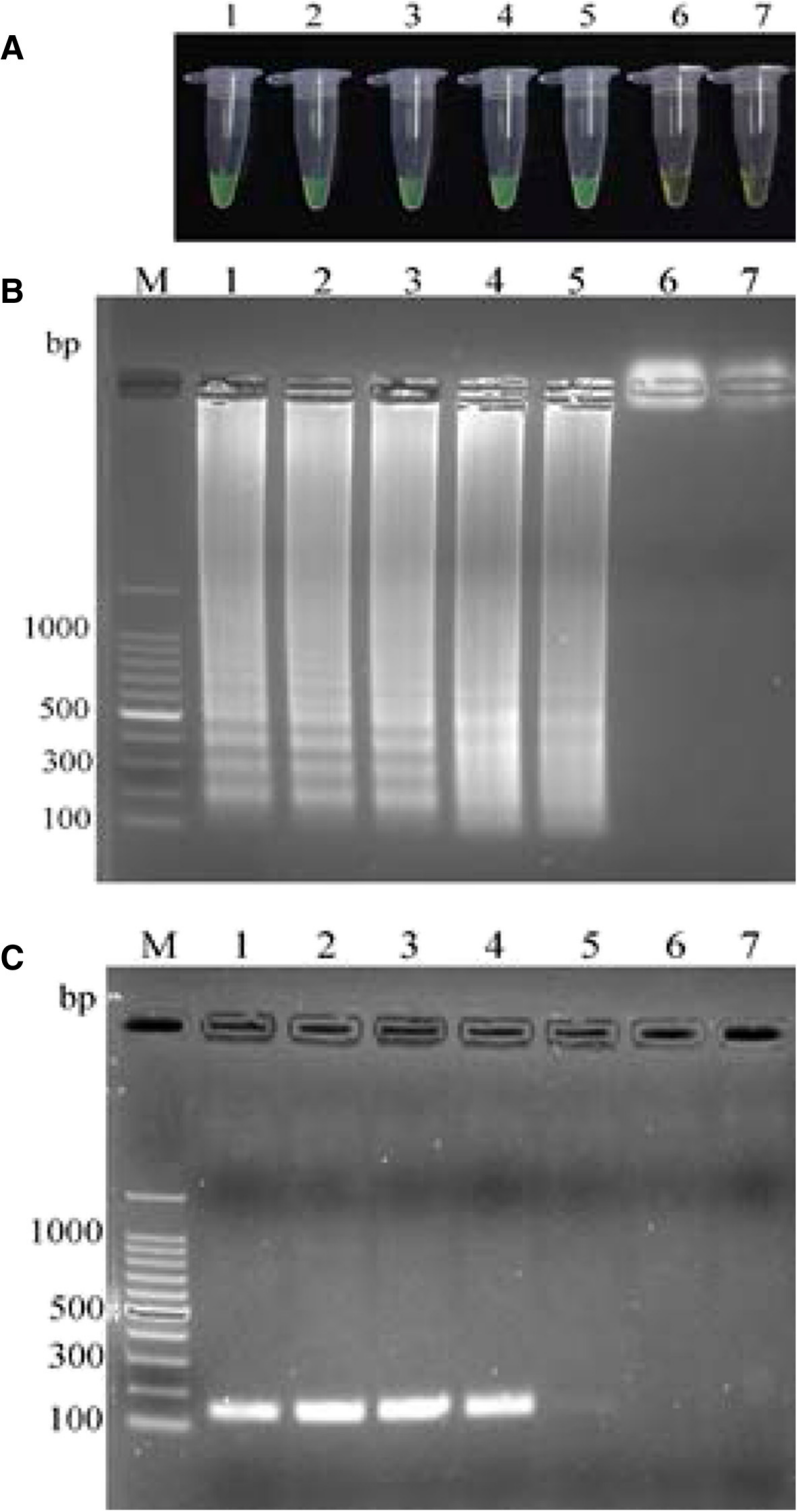
Comparison of detection sensitivity of *L. infantum* LAMP and PCR. Total DNA from *L. infantum* MCAN/CN/90/SC was serially diluted from 10 pg to 0.1 fg and amplified by LAMP and conventional PCR. **a** Visual appearance of LAMP products. **b** Agarose gel electrophoresis of LAMP products. **c** Agarose gel electrophoresis of PCR products. Lane M, 100-bp ladder; lanes 1–6, 10 pg, 1 pg, 100 fg, 10 fg, 1 fg and 0.1 fg DNA, respectively; lane 7, water (negative control)

A set of oligonucleotide primers designed for LAMP reaction of *L. infantum* MCAN/CN/90/SC kDNA minicircle amplified the targeted sequences (Fig. 2). All serial dilutions of the DNA with the exception of that containing 0.1 fg DNA or just water, turned green under the UV light indicating that the detection limit of the assay was 1 fg DNA (Fig. 2a). The presence of LAMP products in the reaction mixture was confirmed by analyzing the reaction products by agarose gel electrophoresis revealing a characteristic pattern of a ladder of multiple bands (Fig. 2b). In contrast, the detection limit of a conventional PCR test using *Leishmania* genus-specific primers was 10 fg DNA, ten times higher than that of the LAMP assay (Fig. 2c).

### Specificity of *L. infantum* LAMP assay

**Fig. 3:**
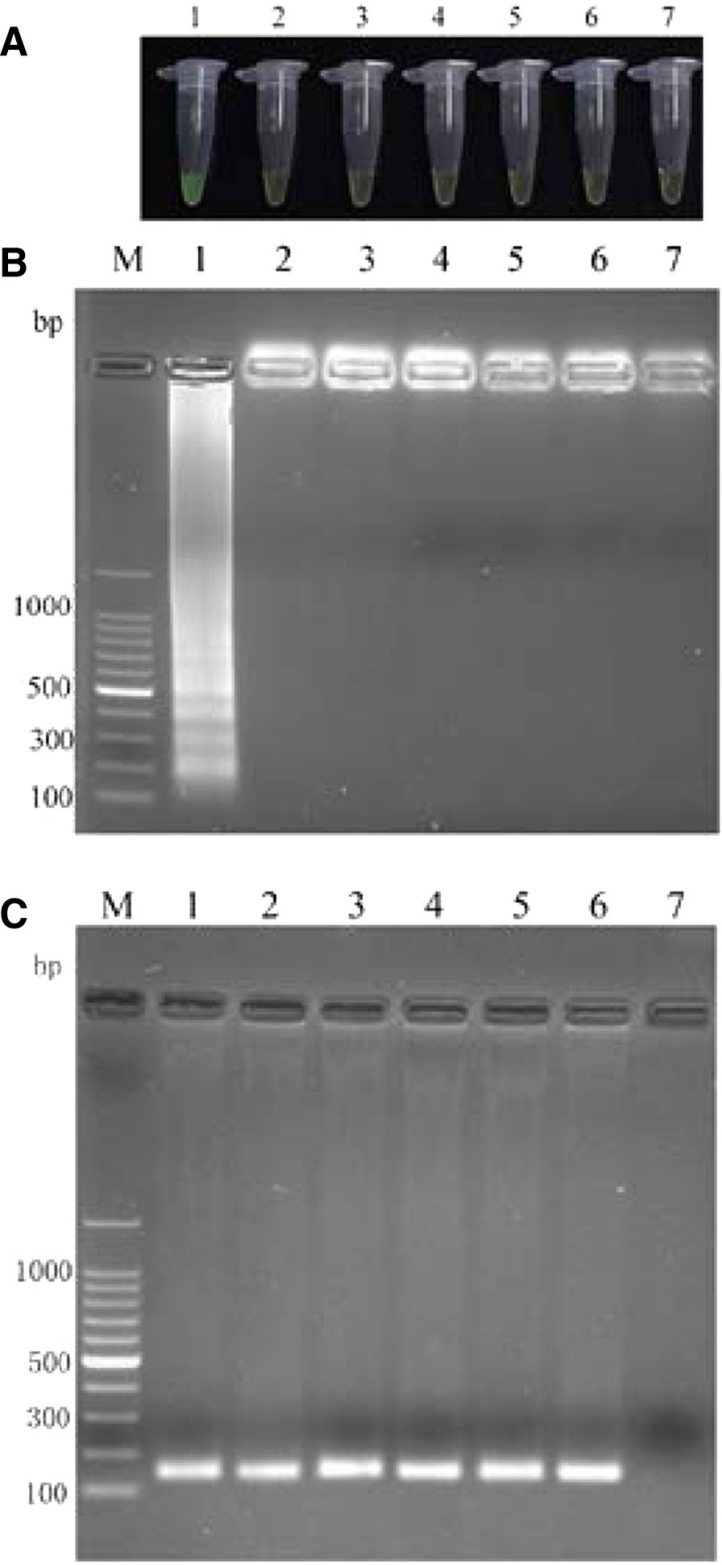
Specificity of *L. infantum* LAMP. Total DNA (100 ng) from different *Leishmania* species and strains were amplified by LAMP using primers targeting *L. infantum* MCAN/CN/90/SC kinetoplast minicircle DNA and by conventional PCR using *Leishmania* genus-specific primers. **a** Visual appearance of LAMP products. **b** Agarose gel electrophoresis of LAMP products. **c** Agarose gel electrophoresis of PCR products. Lane M, 100 bp ladder; lane 1, *L. infantum* MCAN/CN/90/SC; lane 2, *L. donovani* MHOM/IN/80/DD8; lane 3, *L. major* MHOM/SU/75/5ASKH; lane 4, *L. tropica* MHOM/SU/73/K27; lane 5, *L. braziliensis* MHOM/BR/75/M2903; lane 6, *L. infantum* MHOM/TN/80/IPT1; lane 7, water (negative control)

When 100 ng DNA isolated from cultured promastigotes of different *Leishmania* species was used, all reactions remained colorless and no amplification product was detected by agarose gel electrophoresis (Fig. 3a and b). Even a DNA sample from a different *L. infantum* strains (MHOM/TN/80/IPT1) included in the study to determine whether the primers were highly specific for the *L. infantum* isolate of Chinese origin gave a negative result (Fig. 3a and b). However, all samples gave positive results when subjected to conventional PCR using *Leishmania* genus-specific primers confirming that DNA was present in the samples (Fig. 3c).

### Performance of *L. infantum* LAMP assay

Different tissue samples from 111 dogs from the Wudu district (endemic for ZVL) were analyzed by LAMP and PCR (conjunctional swab samples), ELISA (serum samples) and microscopy (bone marrow samples). Only 12 dogs were demonstrated to be positive by all four methods (Table 3). These included 10 samples from dogs with clinical symptoms of VL. Additionally, 31 samples were positive by LAMP, PCR and ELISA, 22 samples by LAMP and PCR, and 3 samples only by LAMP, respectively (Table 3). No samples were positive only by the PCR test or by microscopy. Two samples from asymptomatic dogs were positive by ELISA but not by any other method (Table 3). Forty-one samples were negative by all four methods (Table 3).

**Table 3:** Number of field collected samples from dogs living in the Wudu and Zhang Xian district shown to be positive (+) or negative (−) by LAMP, PCR, ELISA and microscopy (LM)

Region	Number of samples	Assay outcomes			
		LAMP	PCR	ELISA	LM
Wudu	12^a^	+	+	+	+
	31	+	+	+	−
	22	+	+	−	−
	3	+	−	−	−
	2	−	−	+	−
	41	−	−	−	−
	Total 111 (+/−)	68/43	65/46	45/66	12/99
Zhang Xian	1	+	+	−	n.d.^b^
	32	−	−	−	n.d.
	Total 33 (+/−)	1/32	1/32	0/33	n.d.

^a^10 out of the 12 samples were from symptomatic dogs

^b^not determined

Of the 111 dogs examined, 61.3 % (68 dogs (66 in left eye and 65 in right eye)) tested positive for the presence of *L. infantum* using the LAMP assay, 58.6 % (65 dogs (63 in left eye and 61 in right eye)) using the PCR test, 40.5 % (45 dogs) using ELISA and 10.8 % (12 dogs) using microscopy (Table 3). The difference in sensitivity between the LAMP assay and the PCR test was, however, not statistically significant (*χ*^2^ = 0.169, *p* = 0.6810). On the other hand, the sensitivity of the LAMP assay, the PCR test and the ELISA were all extremely significantly different from that of the microscopic examination (*χ*^2^ = 61.285, *p* < 0.0001, *χ*^2^ = 55.853, *p* < 0.0001 and *χ*^2^ = 25.705, *p* < 0.0001, respectively). Both LAMP assay and PCR test were statistically significantly different from the ELISA (*χ*^2^ = 9.535, *p* = 0.0020 and *χ*^2^ = 7.208, *p* = 0.0073).

All of the 33 dogs from the Zhang Xian County tested negative in the ELISA (Table 3). However, one of these dogs tested positive for the presence of leishmanial DNA by both the LAMP assay and the PCR test (Table 3). Based on this, the observed true negative rate (specificity) of both the LAMP assay and the PCR test was 97.0 %.

## Discussion

LAMP is a relatively new nucleic acid amplification method [25], which has been successfully applied as a useful tool in the diagnosis of parasitic infections including human African sleeping sickness and malaria [26, 27]. As the LAMP reaction is a simple and rapid DNA copying procedure that does not require a denaturation step during amplification, the test is particularly useful for diagnosis of infections in the field.

The LAMP primers used in this study amplified a specific target sequence from kDNA minicircles of the *L. infantum* strain MCAN/CN/90/SC but not from kDNA minicircles of other *Leishmania* species. Even with a different *L. infantum* strain (MHOM/TN/80/IPT1) a negative result was obtained. This finding indicates that the LAMP primers were only useful for the detection of Chinese *L. infantum* strains. Similar geographic restrictions have also been reported for PCR primers. For example, PCR primers amplifying a 204 bp fragment from *L. donovani* kDNA minicircles have been shown to be highly specific for *L. donovani* isolates of Indian origin but not for *L. donovani* strains from other parts of the world [28]. However, as the LAMP primers used in this study only identified one strain of *L. infantum* and not many is not a disadvantage per se, as MCAN/CN/90/SC is the only strain in China to infect dogs to cause canine VL. In addition it is very difficult to design universal LAMP primers that can amplify kDNA minicircles from different strains of *L. infantum* because there are only a few short sequence stretches of kDNA minicircles that are common between different *Leishmania* strains [29–31].

Accurate and rapid diagnosis of canine leishmaniasis is essential to reduce the number of infected dogs in endemic areas in order to prevent the transmission of the disease from the animal reservoir to humans. Although various methods are available for detection of *Leishmania* spp. in dogs, molecular techniques have typically much greater sensitivity than parasitological or serological tests. The LAMP assay developed in this study had a detection limit of 1 fg of *L. infantum* DNA purified from cultured promastigotes; an amount that represents an equivalent of about 0.1 parasites. This high sensitivity was achieved by targeting kDNA minicircles, of which, thousands of copies are present in a trypanosomatid cell [32]. This detection limit is 50 times lower than that of a recently developed *L. infantum* LAMP assay targeting the cysteine protease B multi copy gene [33].

LAMP assays have been previously shown to detect *Leishmania* infections with high sensitivity and specificity when using blood samples [33–36]. However, like conventional PCR, LAMP is also sensitive to various substances present in biological fluids, although LAMP is less affected by these components than the PCR [37]. In addition, to make the LAMP technique feasible for field use, the sampling method needs to be easy, non-invasive and painless; conjunctival swabs as a sampling method fulfills all of these requirements. While DNA extraction and purification from blood samples is usually required for nucleic acid amplification methods, conjunctival swabs can just be boiled and centrifuged in order to extract the DNA. Moreover, conjunctival swab sampling in connection with conventional PCR has been shown to be highly sensitive in the detection of *Leishmania* infections in sick or subclinically infected dogs [21, 38–40]. Equally important is the fact that non-invasive sample collection is also more acceptable to dog owners.

In the absence of a gold standard (note that serological and parasitological methods are not very sensitive in the diagnosis of subclinical leishmaniasis in dogs [13, 20]), the true positive rate (sensitivity) of the LAMP assay could not be determined. However, the newly developed LAMP assay appeared to be more sensitive than conventional PCR, ELISA and microscopy, as more dogs were diagnosed positive for infection with *L. infantum*. That the LAMP method achieves higher positive detection rates than PCR tests is not uncommon and has been reported for other parasitic diseases [41, 42].

The observed true negative rate (specificity) of the newly developed LAMP assay (97 %) is comparable with the specificities of other LAMP assays designed for the detection of VL in blood samples (80-98 % [33, 35]). Only one out of 33 dogs from a non-endemic area of ZVL gave a false positive result. The reason for this is unclear. It could be due to contamination of the DNA sample extracted from the conjunctival swab, as the PCR test was also positive with this sample.

## Conclusion

In summary, a simple and rapid non-invasive conjunctival swab LAMP assay for the sensitive and specific detection of *L. infantum* in dogs was developed. As this test is an affordable and easily applied method, it is useful for epidemiological surveillance of *L. infantum* infections in dogs in order to control ZVL in endemic areas in western China.
